# Skeletal Muscle Diffuse Large B-Cell Lymphoma in the Gluteal Region

**DOI:** 10.4274/tjh.2018.0186

**Published:** 2018-11-13

**Authors:** Nereyda Gonzalez-Benavides, Jesus Alberto Cardenas-de la Garza, Candelario Rodriguez-Vivian, Jorge Ocampo-Candiani, Oliverio Welsh

**Affiliations:** 1 Autonomous University of Nuevo León, Dr. Jose E. Gonzalez Faculty of Medicine and University Hospital, Department of Dermatology, Monterrey, Mexico

**Keywords:** Diffuse large B-cell lymphoma, Extranodal lymphoma, Gluteal lymphoma, Muscle lymphoma

## To the Editor,

Diffuse large B-cell lymphoma (DLBCL) is the most common form of non-Hodgkin lymphoma (NHL) [1]. Approximately 30% of NHL cases arise from an extranodal site, including the skin, testes, lungs, bones, gastrointestinal tract, and central nervous system [1,2]. Primary skeletal muscle lymphomas are rare and account for 0.5% of NHL cases [3].

A 60-year-old male presented with a 5-month history of a rapidly growing mass in his left buttock accompanied by intense pain and impaired mobilization. He denied weight loss, fever, or night sweats. Physical examination revealed a firm, tender left buttock mass, measuring 19x13 cm (Figure 1a). No palpable lymph nodes were detected. Laboratory tests were unremarkable. Abdominal and pelvic contrast-enhanced CT scan showed a soft tissue tumor in the left gluteal region, affecting the psoas, gluteus maximus, and minor muscles with left retroperitoneal and inguinal lymphadenopathy. Two deep punch biopsies were performed. Histopathological examination revealed diffuse atypical lymphocyte infiltration involving the dermis, subcutaneous tissue, and muscle. Immunohistochemical staining was positive for CD20, with focal positivity of 20% for MUM1, and negative for CD10 and BCL6. The Ki-67 proliferation index was 80%. The final diagnosis was DLBCL, activated B-cell subtype. Six cycles of chemotherapy with rituximab, cyclophosphamide, doxorubicin, vincristine, and prednisone (R-CHOP) were started. He obtained complete clinical remission (Figure 1b) with no recurrence.

Extranodal lymphomas (ENLs) are defined as those with no/minimal nodal involvement associated with a dominant extranodal component [4]. However, the definition of primary lymphoma remains a controversial issue, especially in patients where both nodal and extranodal sites are involved. The Lugano classification designates extranodal disease as single extranodal lesions without nodal involvement or patients with state I or II nodal disease with a clinically dominant extranodal component [5,6,7]. ENLs may arise from any site devoid of lymphocytes and almost half represent DLBCL [6].

Involvement of the skeletal muscles in NHL is unusual and has been reported to occur in 1.1% of patients. The most common route of muscle involvement is hematogenous, lymphatic, or by contiguous spread, or, very rarely, as a primary extranodal disease [4]. The most commonly affected muscles are those of the extremities, pelvis, and gluteal regions [6]. In a retrospective study from the Mayo Clinic of over 7000 cases of lymphoma, primary muscle lymphoma accounted for only 0.1%, as diagnosed over a 10-year period [8].

The main symptoms include the presence of a mass with progressive enlargement, pain, and swelling [9]. Imaging studies show diffuse enlargement of the muscle involving multiple compartments, distinguishing it from soft tissue sarcomas that usually involve one compartment [9]. Magnetic resonance imaging may aid in diagnosis and enables evaluation of tumor extension and adjacent structure involvement. However, histological analysis and immunohistochemistry is necessary to confirm the diagnosis [10].

Differential diagnosis includes soft tissue sarcoma, metastatic carcinoma, and neurogenic tumors such as malignant peripheral nerve sheath tumors [6]. No specific guidelines for the treatment of skeletal muscle ENLs are available. R-CHOP chemotherapy is usually the preferred regimen [7]. Due to the scarce number of reports, information on the precise prognosis of primary skeletal ENLs is not available.

## Figures and Tables

**Figure 1a:**
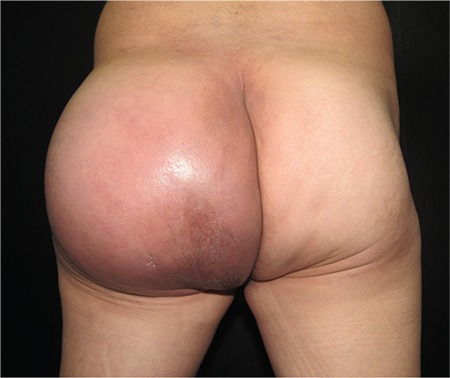
Diffuse large B-cell lymphoma in the gluteal region before treatment.

**Figure 1b:**
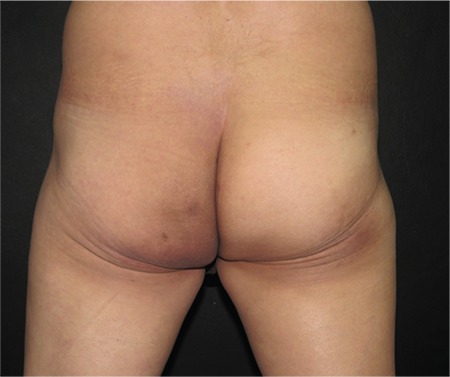
After 6 cycles of R-CHOP chemotherapy.
